# Supplementary material for: Biologging as an important tool to uncover behaviors of cryptic species: an analysis of giant armadillos (Priodontes maximus)
Source: PeerJ. 2023 Jan 18;11:e14726. doi: 10.7717/peerj.14726 (PMC9864128; doi:10.7717/peerj.14726)
Supplement: Supplemental Information 1 [file peerj-11-14726-s001.docx]

SUPPLEMENTARY MATERIAL

**Biologging as an important tool to uncover behaviors of cryptic species: A case-study of giant armadillos (*Priodontes maximus*)**

Joshua A. Cullen^a,b^, Nina Attias^c,d^, Arnaud L.J. Desbiez^c,e,f^, Denis Valle^a^

^a^School of Forest, Fisheries, and Geomatics Sciences, University of Florida, Gainesville, FL, USA

^b^Department of Earth, Ocean and Atmospheric Science, Florida State University, Tallahassee, FL, USA

^c^Instituto de Conservação de Animais Silvestres (ICAS), Campo Grande, Mato Grosso do Sul, Brazil

^d^Department of Wildlife Ecology & Conservation, University of Florida, Gainesville, FL, USA

^e^Instituto de Pesquisas Ecológicas (IPÊ), Nazaré Paulista, São Paulo, Brazil

^f^Royal Zoological Society of Scotland (RZSS), Murrayfield, Edinburgh, United Kingdom


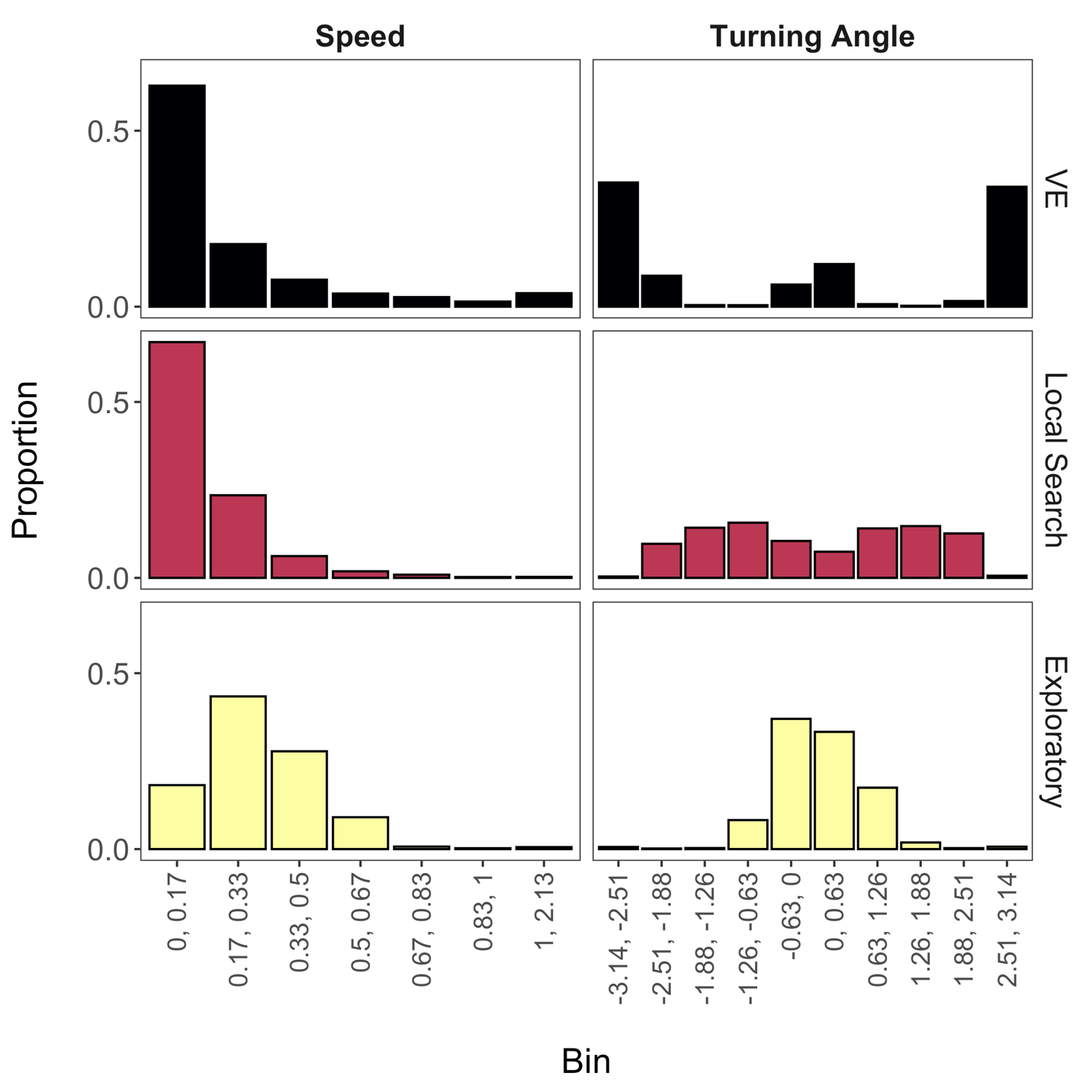


**Figure S1** State-dependent distributions estimated by the mixture model for speed and turning angle (only) are shown for each of the three identified behavioral states. The limits (min, max) associated with each bin are shown for speed (m/s) and turning angles (radians) on the x-axis. The vigilance-excavation state is abbreviated as ‘VE’.


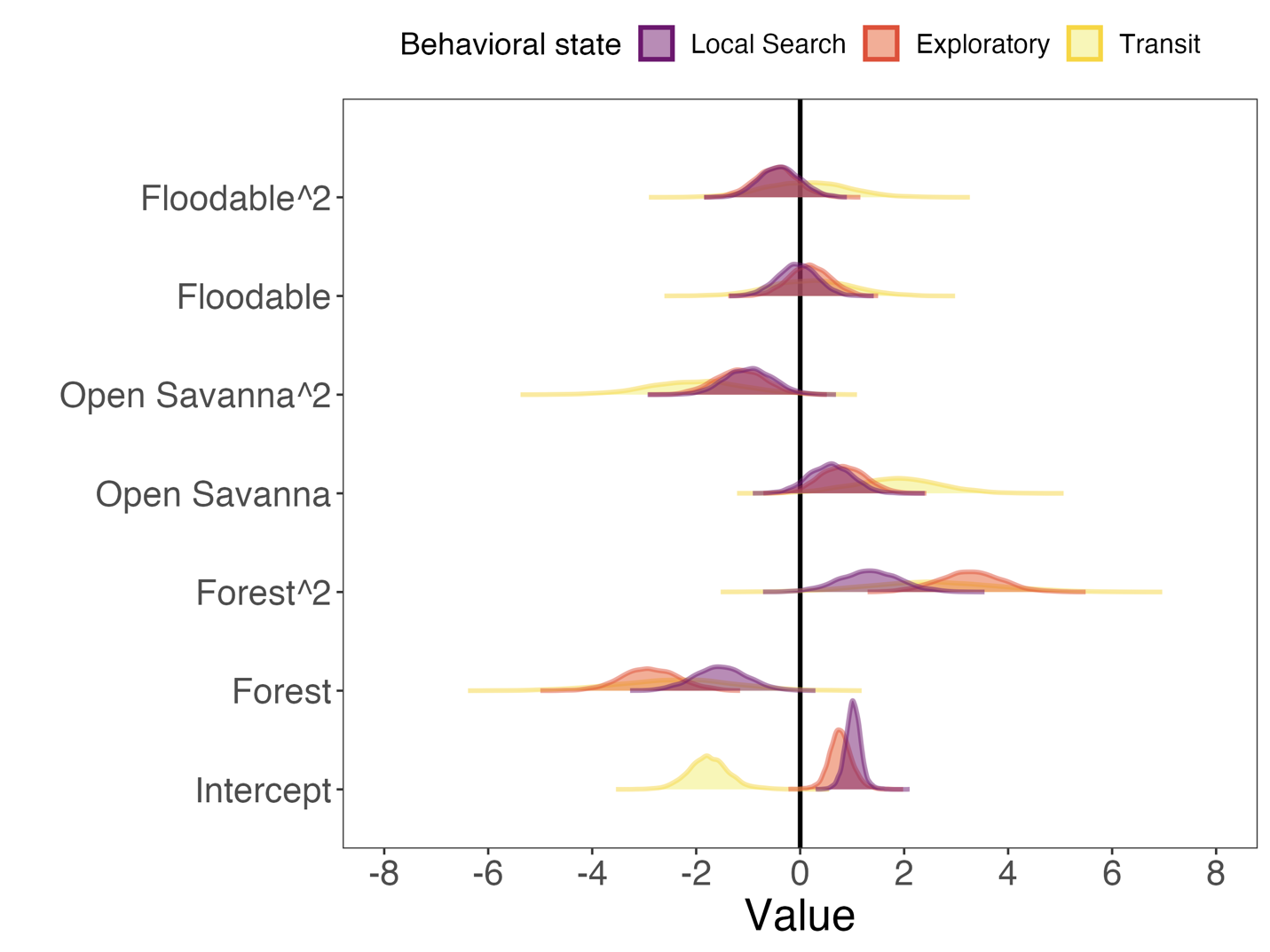


***Figure S2*** *Posterior density distributions of parameter estimates from the multinomial logistic regression that relates proportions of land cover classes to behavioral states. The vertical line denotes a value of zero to clarify the direction and magnitude of parameter estimates. Closed Savanna serves as the reference class for the explanatory variable of this model and is therefore not estimated.*


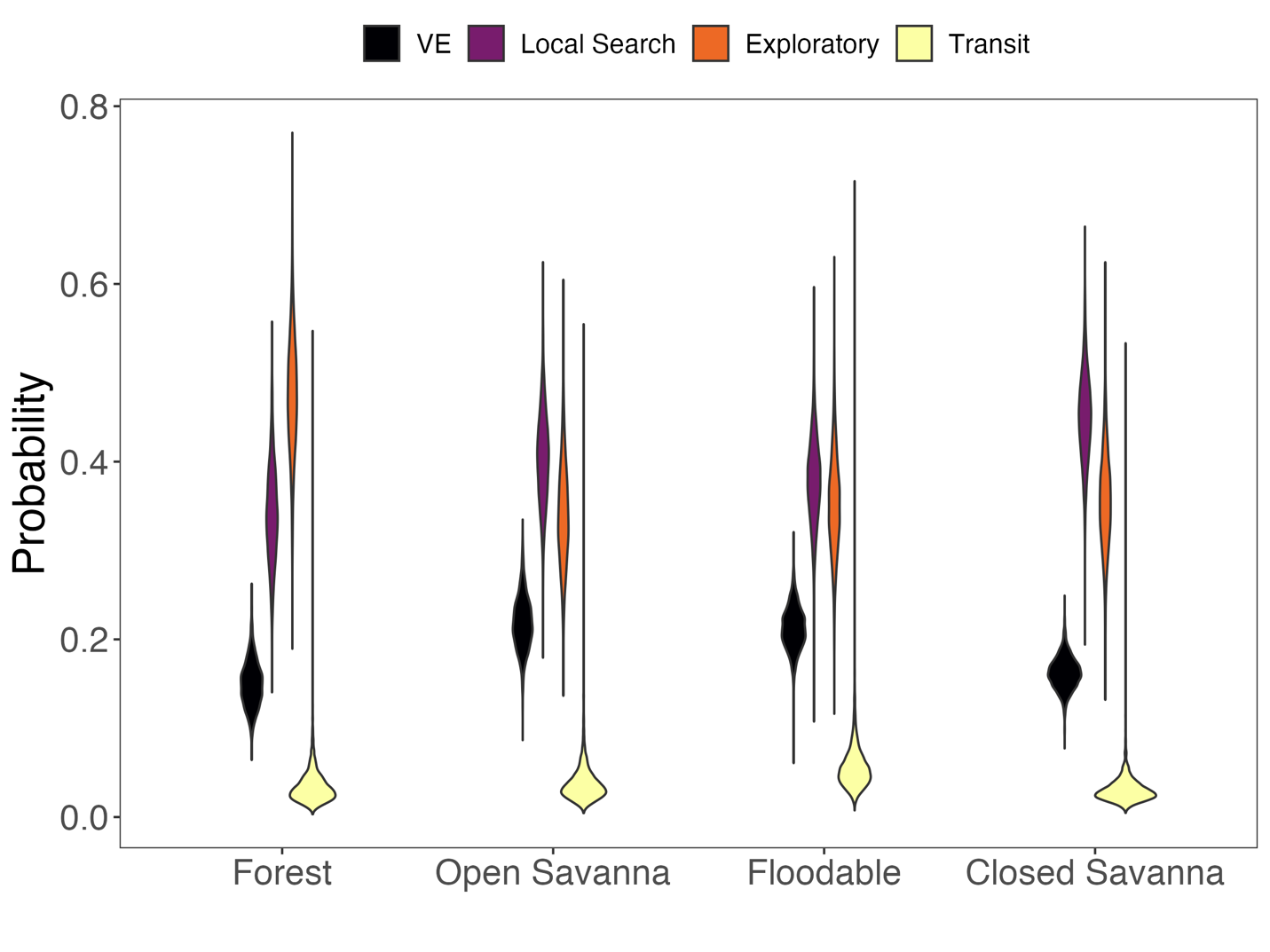


**Figure S3** Estimated probabilities of exhibiting each behavioral state when in 100% of a given land cover class as predicted by the multinomial logistic regression. The Closed Savanna class is also shown since it is the equivalent of setting all other land cover classes to 0%. The vigilance-excavation state is abbreviated as ‘VE’.

Table S1. Model summary from the Bayesian multinomial logistic regression that relates proportion of land cover classes to each of the three estimated behavioral states. Notice that vigilance-excavation (VE) is the reference behavioral state and, as a result, is not included in this table. Additionally, Closed Savanna serves as the reference class for the explanatory variable of this model and is therefore not estimated. Squared terms denote the quadratic effect of each land cover class within the model. Estimates are reported on the multinomial logit scale. Bolded rows indicate the coefficients where the 95% credible interval did not contain zero.

| State | Variable | Coefficient | Standard Error | 95% Credible Interval |
| --- | --- | --- | --- | --- |
| Local Search | **Intercept** | **1.02** | **0.15** | **(0.72, 1.33)** |
|  | **Forest** | **-1.54** | **0.53** | **(-2.61, -0.52)** |
|  | **Forest^2^** | **1.34** | **0.61** | **(0.18, 2.53)** |
|  | Open Savanna | 0.58 | 0.42 | (-0.25, 1.41) |
|  | **Open Savanna^2^** | **-0.99** | **0.46** | **(-1.89, -0.06)** |
|  | Floodable | -0.06 | 0.40 | (-0.83, 0.74) |
|  | Floodable^2^ | -0.38 | 0.41 | (-1.21, 0.41) |
| Exploratory | **Intercept** | **0.78** | **0.23** | **(0.33,1.23)** |
|  | **Forest** | **-2.89** | **0.56** | **(-3.98, -1.79)** |
|  | **Forest^2^** | **3.27** | **0.63** | **(2.02, 4.51)** |
|  | Open Savanna | 0.82 | 0.44 | (-0.04, 1.71) |
|  | **Open Savanna^2^** | **-1.16** | **0.49** | **(-2.14, -0.22)** |
|  | Floodable | 0.18 | 0.41 | (-0.66, 0.98) |
|  | Floodable^2^ | -0.46 | 0.43 | (-1.29, 0.41) |
| Transit | **Intercept** | **-1.74** | **0.42** | **(-2.54, -0.92)** |
|  | **Forest** | **-2.39** | **1.08** | **(-4.52, -0.38)** |
|  | **Forest^2^** | **2.54** | **1.21** | **(0.20, 4.93)** |
|  | **Open Savanna** | **1.83** | **0.86** | **(0.22, 3.50)** |
|  | **Open Savanna^2^** | **-1.96** | **0.96** | **(-3.82, -0.14)** |
|  | Floodable | 0.23 | 0.79 | (-1.37, 1.71) |
|  | Floodable^2^ | 0.11 | 0.81 | (-1.44, 1.77) |
